# Regulation of Lipid Signaling by Diacylglycerol Kinases during T Cell Development and Function

**DOI:** 10.3389/fimmu.2013.00178

**Published:** 2013-07-04

**Authors:** Sruti Krishna, Xiao-Ping Zhong

**Affiliations:** ^1^Department of Pediatrics, Division of Allergy and Immunology, Duke University Medical Center, Durham, NC, USA; ^2^Department of Immunology, Duke University Medical Center, Durham, NC, USA

**Keywords:** diacylglycerol kinase, phosphatidic acid, T cell development, T cell activation, T cell tolerance, T cell receptor, mast cells, macrophages

## Abstract

Diacylglycerol (DAG) and phosphatidic acid (PA) are bioactive lipids synthesized when the T cell receptor binds to a cognate peptide-MHC complex. DAG triggers signaling by recruiting Ras guanyl-releasing protein 1, PKCθ, and other effectors, whereas PA binds to effector molecules that include mechanistic target of rapamycin, Src homology region 2 domain-containing phosphatase 1, and Raf1. While DAG-mediated pathways have been shown to play vital roles in T cell development and function, the importance of PA-mediated signals remains less clear. The diacylglycerol kinase (DGK) family of enzymes phosphorylates DAG to produce PA, serving as a molecular switch that regulates the relative levels of these critical second messengers. Two DGK isoforms, α and ζ, are predominantly expressed in T lineage cells and play an important role in conventional αβ T cell development. In mature T cells, the activity of these DGK isoforms aids in the maintenance of self-tolerance by preventing T cell hyper-activation and promoting T cell anergy. In this review, we discuss the roles of DAG-mediated pathways, PA-effectors, and DGKs in T cell development and function. We also highlight recent work that has uncovered previously unappreciated roles for DGK activity, for instance in invariant NKT cell development, anti-tumor and anti-viral CD8 responses, and the directional secretion of soluble effectors.

## Introduction

Lipids are small hydrophobic molecules that perform a variety of cellular functions. Though best known for their role in maintaining cell structure and storing energy, lipids have gained in importance over the past few decades as signaling mediators (1, 2). While lipids that participate in signaling are thought to be much less abundant in the cell as compared to structural lipids, their levels vary dynamically in response to external signals.

In this review, we discuss the signaling functions of two key lipid second messengers, diacylglycerol (DAG) and phosphatidic acid (PA), in the context of T cell development and function. DAGs are esters of glycerol in which two of its hydroxyl groups are esterified with long-chain fatty acids. One manner of PA generation in cells is via phosphorylation of the free hydroxyl group in DAG by a family of enzymes called diacylglycerol kinases (DGKs) (3, 4). DGKs therefore act as molecular switches that simultaneously turn off DAG-mediated signaling and turn on PA-mediated signals.

While all 10 mammalian DGK isoforms contain a kinase domain and at least two cysteine-rich C1 domains, they can be grouped into five types based on the homology of their other structural features. α and ζ are the major isoforms expressed in T cells (5). DGKα is a type I DGK and contains an N-terminal recoverin homology domain and two Ca2^+^-binding EF hands. DGKζ is a type IV DGK and contains a myristoylated alanine rich C kinase substrate (MARCKS) motif, four ankyrin repeats and a C-terminal PDZ-binding domain. DGKζ undergoes alternative splicing, producing a 130 kDa ζ2 isoform that is highly expressed in immature thymocytes and a 115 kDa ζ1 isoform that is predominant in mature thymocytes and peripheral T cells (6). Functional differences between the two splice variants remain unclear.

Here, we begin by discussing the various effector molecules that transduce DAG-mediated signals and PA-mediated signals. We then switch gears to review our current understanding of the role of DGKs in T cell development and function with an emphasis on recent advances that have revealed hitherto unknown functions for these enzymes. The roles of DGK activity in other immune cell lineages are also discussed briefly.

## DAG-Mediated Signaling

**Figure 1:**
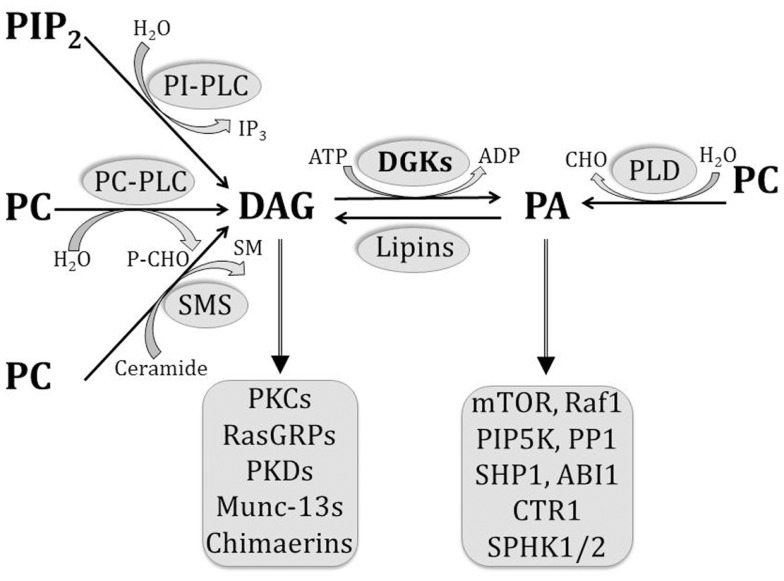
**Pathways involved in the generation and removal of DAG and PA**. Multiple pathways contribute to DAG generation in the cell, including the hydrolysis of PIP_2_ by PI-dependent PLCs, hydrolysis of PC by PC-dependent PLCs, dephosphorylation of PA by lipins, and sphingomyelin synthesis by SMS. On the other hand, PA is generated by PLD-mediated hydrolysis of PC and by DGK-mediated phosphorylation of DAG. As bioactive lipids, both DAG and PA are able to bind to a number of effector molecules, as listed.

Several enzymes contribute toward DAG production upon receptor stimulation in immune cells (7) (Figure 1). Phosphatidylinositol-dependent phospholipases hydrolyze membrane phosphatidylinositol bisphosphate (PIP_2_) to DAG and inositol triphosphate, and phosphatidylcholine (PC)-dependent phospholipases hydrolyze PC to DAG and phosphoryl choline. In addition, sphingomyelin synthase generates DAG and sphingomyelin from PC and ceramide, while PA phosphatases such as lipins dephosphorylate PA to DAG. On the other hand, DAG is primarily removed by the activity of DGKs, which catalyze its phosphorylation to PA. DAG can recruit a variety of downstream effector molecules through their C1 domains, and thereby trigger multiple signaling pathways.

The protein kinase C (PKC) family of serine/threonine kinases consists of 10 isozymes that are activated by a number of distinct mechanisms (8, 9). Upon engagement of the TCR, production of DAG by activated PLCγ1 recruits PKCθ to the plasma membrane in T cells. Co-stimulation via CD28 also plays an important role in the recruitment and spatial segregation of PKCθ at the immunological synapse (10, 11). Activation of PKCθ is indispensable for TCR-mediated NF-κB activation in mature T cells (12, 13). A role for PKCθ has also been identified in an array of key processes (14) including invariant NKT (*i*NKT) cell development and activation (15, 16), T cell survival (17), IL-2 production (18), T_H_2 responses (19, 20), and T_H_17 responses (21). Thus, by recruiting PKCθ, DAG regulates multiple aspects of T cell function.

Another important protein that is brought to the plasma membrane by DAG upon TCR stimulation is Ras guanyl-releasing protein 1 (RasGRP1) (22). RasGRP1 is a member of the RasGRP family of factors that help activate Ras by exchanging bound GDP for GTP (23), and is selectively expressed in T cells and a few other cell types (24). RasGRP1 plays an essential role in thymocyte development (25), and is particularly required for the selection of thymocytes that express weakly selecting TCRs (26). RasGRP1 is not critical for the development of γδ T cells, but is important for their proliferation and IL-17 production (27). Other studies have shown that RasGRP1 may play a role in promoting antigen-induced CD8 cell expansion by lowering the threshold of T cell activation (28). RasGRP1 is therefore a key effector downstream of DAG that plays a multitude of critical roles in T cell development and function.

Members of the protein kinase D (PKD) family have been identified more recently as DAG effectors (29). A unique characteristic of PKDs is that they are targets of both DAG and DAG-activated PKCs (30). PKDs are thought to be activated by a multi-step mechanism. Upon cell stimulation, inactive PKD translocates from the cytosol to the plasma membrane in response to membrane DAG production, where it is then activated by novel PKCs that are also recruited to the membrane by DAG. During T cell development, PKD has been shown to exert different effects on VDJ recombination at the TCRβ locus and on CD4 and CD8 expression, based on its localization at the cytosol or plasma membrane (31). Subsequent work has revealed that regulation of thymocyte development by membrane-localized PKD, but not cytosol-localized PKD, is dependent on the GTPase RhoA (32). Bringing PKD to the plasma membrane therefore represents another important mechanism by which DAG regulates T cell development.

Munc13 proteins are mammalian homologs of the *C. elegans* Unc13 that is localized to pre-synaptic active zones of neurons and important for neurotransmitter secretion (33). Munc13-1, Munc13-2, and Munc13-3 isoforms bind to DAG with high affinity, and translocate to the plasma membrane in response to receptor stimulation. In the immune system, the Munc13-4 isoform which lacks a C1 domain (34, 35) has been shown to be important for granule maturation and exocytosis in NK cells and cytotoxic T lymphocytes (CTLs) (36, 37), and for phagosomal maturation and killing of intracellular bacteria in neutrophils (38, 39). Further studies are required to investigate parallel roles for DAG-binding Munc13 isoforms in NK cells, CTLs, neutrophils, and other types of immune cells. Over-expressing human Munc13 in opossum renal epithelial cell lines enhanced their susceptibility to apoptosis after DAG treatment, suggesting that Munc13 proteins may transduce apoptosis-inducing signals downstream of DAG in some cell types (40). The role of Munc13 proteins in T cell development and function remain poorly understood.

Chimaerins, a family of proteins that possess Rac-specific GTPase Activating Protein (GAP) activity, contain C1 domains that bear about 40 percent homology to those of PKCs (41, 42). Chimaerin isoforms α2 and β2 are expressed at different levels in T cells and have been shown to participate in TCR signaling (43). Results from the study suggest that these chimaerin isoforms translocate to the immunological synapse upon T cell activation, but in a manner that is independent of canonical DAG-binding by the C1 domains. Catalytic activity of these chimaerins was found to play an important role in inhibiting TCR-mediated NFAT activation. Other studies have delineated a role for β2 chimaerin in mediating DAG-dependent changes in T cell adhesion and chemotaxis (44). In this study, expression of GFP-tagged β2 chimaerin revealed that active Rac and C1-dependent PMA binding could co-operate to induce sustained localization of β2 chimaerin to the plasma membrane in Jurkat T cells. Overexpression of GFP-β2 chimaerin was associated with decreased CXCL12-induced static adhesion but enhanced CXCL12-induced migration. Chimaerin proteins therefore represent an important class of DAG effectors in T cells, but further work is required to dissect aspects of their function that are dependent on and independent of DAG-binding.

## PA-Mediated Signaling

Diacylglycerol kinases and enzymes of the phospholipase D (PLDs) family act as key mediators of PA production in immune cells by phosphorylating DAG and hydrolyzing PC, respectively (7, 45) (Figure 1). On the other hand, enzymes such as lipins that possess PA phosphatase activity play a critical role in turning off PA-mediated signaling by removing PA (46). Cellular levels of PA have been shown to change dynamically in response to environmental stimuli, and a wealth of data has revealed a diverse array of functions for this bioactive lipid (47).

Phosphatidic acid performs its signaling functions primarily by associating with a growing number of effector molecules that include kinases such as mammalian/mechanistic target of rapamycin (mTOR) and phosphatidylinositol-4-phosphate 5-kinase (PIP5K), and phosphatases such as Src homology region 2 domain-containing phosphatase 1 (SHP1) (48). In mammalian HEK293 cells, treatment with exogenous PA was found to promote the phosphorylation of S6K1 and 4E-BP1, which are substrates of mTOR complex 1 (49). This phosphorylation was abolished by rapamycin, a bacterial macrolide that inhibits mTOR activity. Results from this study showed that mitogenic stimulation of HEK293 cells led to cellular PA accumulation within 5 min. Small unilamellar vesicles containing PA could also directly bind to the FKBP12-rapamycin binding (FRB) domain on mTOR in a manner that competed with FKBP12-rapamycin. Together, these results suggest a role for PA as a critical mediator that connects mitogenic stimuli to mTOR activation in mammalian cells (50). More recent work has revealed that PA may activate mTOR by a distinctive two-step mechanism that involves the displacement of the endogenous mTOR-inhibitor FKBP38 and allosteric activation of the kinase (51). On the other hand, studies with Rat2 fibroblasts suggest that PA may indirectly activate mTOR complex 1 via the MEK-ERK pathway (52). In this study, two structurally distinct MEK inhibitors were found to inhibit PA-mediated activation of mTOR complex 1. Other studies with human renal cell adenocarcinoma cell lines have shown that suppression of cellular PA production by treatment with a PLD inhibitor may inhibit the association of mTOR with both Raptor and Rictor, suggesting that PLD-derived PA may act as a key stabilizer of mTOR complexes 1 and 2 (53).

Diacylglycerol kinase-derived PA has also been shown to modulate mTOR complex 1 activity in HEK293 cell lines (54). Overexpression of DGKζ, but not DGKα, led to increased mTOR complex 1-dependent phosphorylation of S6K1 both in the presence and absence of serum, suggesting that DGKζ-derived PA may activate mTOR complex 1. DGKζ-induced S6K1 phosphorylation was dose-dependent, as cells expressing higher levels of DGKζ showed more intense S6K1 phosphorylation. DGKζ-mediated increase in S6K1 phosphorylation was abolished when a mutant form of mTOR (that lacked the capacity to bind PA) was co-expressed, suggesting that DGKζ-generated PA enhances S6K1 phosphorylation by directly binding to mTOR.

While it is tempting to extend these observations to suggest that PA may directly bind and activate mTOR in immune cells, only a few studies have rigorously examined the relationship between PA and mTOR in these cell types. RAW264.7 macrophage cell lines showed enhanced secretion of pro-inflammatory cytokines such as IL-6, TNF-α, and IL-1β in response to PA stimulation (55). This was specifically abrogated by rapamycin, suggesting that PA-induced production of pro-inflammatory cytokines in macrophages may occur through the mTOR complex 1 pathway. Treatment of mice with PA also led to an increase in serum levels of these cytokines, suggesting that PA may promote systemic inflammatory responses in a manner that is dependent on mTOR complex 1 activation.

As we discuss later in this review, a bevy of studies provide clear genetic evidence of critical roles for DGKα and DGKζ in multiple aspects of T cell maturation and function. However, much work remains to be done in understanding the role of DGK-derived and PLD-derived PA in activating mTOR complex 1 during T cell development and function.

Another key signaling pathway that is activated by PA is the Ras/MEK/ERK signaling cascade (56). The first line of evidence for PA-mediated modulation of MAP kinase signaling came from a study which showed that a C-terminal domain of Raf1 could bind to PA in a canine kidney cell line (57). Subsequent work showed that growth factor-mediated activation of PLD and the concomitant production of PA were directly linked to the activation of the Raf1-MAP kinase pathway (58). Based on findings that insulin-dependent PLD activation was critically dependent on ADP ribosylation factor (ARF) activation, the researchers used an ARF inhibitor (brefeldin A) to block PLD activation (59). Blocking PLD activity and PA production with brefeldin A blocked insulin-induced activation of the MAPK pathway in Rat-1 fibroblasts. Results from the study also showed that PA did not directly activate Raf1, but instead enhanced its recruitment to the plasma membrane to allow for its activation by Ras. Mutations in Raf1 that disrupted Raf1-PA interaction prevented plasma membrane recruitment of Raf1 in response to insulin stimulation (60).

Kinase suppressor of Ras 1 (KSR1) is a scaffolding protein that interacts with several components of the Raf-MEK-ERK cascade to co-ordinate the formation of localized multi-protein complexes that enable efficient signal transduction (61). More recent work has shown that KSR1 contains a sequence homologous to Raf1’s PA-binding domain that allows it to directly bind PA and be recruited to the plasma membrane in response to insulin stimulation in fibroblasts (62). The recruitment of KSR1 by PA was also found to be essential for its scaffolding function, as mutations in its PA-binding motif impaired insulin-induced MEK and ERK phosphorylation. Studies have shown that PA also recruits Sos, an activator of Ras, to the plasma membrane through its plextrin homology domain (63). PLD2-derived PA was found to play essential role in EGF-induced Ras activation, as mutations of Sos that impaired PA-binding prevented its membrane recruitment and subsequent activation of Ras.

Studies with Jurkat T cells suggest that PLD2 may act upstream of RasGRP1 upon TCR crosslinking and co-stimulation via the integrin lymphocyte function-associated antigen 1 (LFA1) (64). In this case, production of PA by PLD2 and subsequent dephosphorylation of PA to DAG by PA phosphatase were shown to be critical for plasma membrane Ras activation. Others have demonstrated a role for PLD-derived PA in ERK1/2 activation downstream of Galectin-8 engagement in Jurkat T cells (65). Galectins are a family of widely expressed carbohydrate-recognizing proteins, and Gal-8 has previously been shown to bind certain integrins on the T cell surface to provide co-stimulatory and proliferative signals (66). In this study, Gal-8 induced PA accumulation in Jurkat T cells within 15 min in a manner that was inhibited by treatment with a PLD inhibitor (1-butanol). Such PLD-inhibition also abrogated ERK1/2 phosphorylation, suggesting that PLD-derived PA may play an essential role in ERK activation downstream of Gal-8 stimulation in Jurkat T cells. However, this result must be interpreted with caution, as PLD-derived PA may also activate ERK in an indirect manner involving the lipin-mediated conversion of PA to DAG.

Type I PIP5K enzymes catalyze the production of PIP_2_ from phosphatidylinositol-4-phosphate (PIP). During T cell activation, PIP_2_ is involved in modulating T cell rigidity but is primarily hydrolyzed to produce key second messengers DAG and inositol triphosphate (IP_3_) (51). A number of studies have established that PA can bind to and activate PIP5K (67). Modulation of PIP5K activity by PA was originally shown using PIP5K purified from bovine brain membranes, where PA was shown to enhance enzymatic activity by up to 20-fold (68). Subsequent studies demonstrated that type I PIP5Ks (which phosphorylate PIP to PIP_2_), but not type II PIP5Ks (which phosphorylate phosphatidylinositol 5 phosphate to PIP_2_), are specifically regulated by PA (69). More recent work has suggested that PA may regulate the affinity of murine PIP5K-1β for its substrate PIP (70). In this study, PA was shown to bind specifically to the C-terminal region of PIP5K-1β and kinetic analysis revealed that the addition of PA increased the affinity of PIP-binding to the enzyme’s active site by nearly 70-fold. Other studies have elegantly demonstrated a role for DGKζ-derived PA in stimulating PIP5K-1α activity to increase local PIP_2_ levels and promote actin polymerization in cell lines (71). Expression of DGKζ enhanced PIP5K-1α activity in thrombin-stimulated HEK293 cells, and DGKζ and PIP5K-1α were found to co-localize and co-immunoprecipitate with each other. DGKζ and PIP5K-1α were also found to co-localize with actin at lamellipodial protrusions in epithelial cells. While there is evidence to suggest that the activity of PIP5K-1α and γ isoforms may be critical for normal human NK cell cytotoxicity (72, 73), the role of PLD-derived and DGK-derived PA in regulating PIP5K activity in immune cells remains quite poorly understood.

Protein phosphatase 1 (PP1) is a eukaryotic serine/threonine phosphatase that regulates the function of a variety of proteins in the cell. The PP1 catalytic subunit is able to interact with more than 50 different regulatory subunits in a mutually exclusive manner and this allows the enzyme to target different substrates in diverse subcellular locations depending on its binding partner (74). Initial studies identified that PA acted as a highly specific tight-binding inhibitor of the γ isoform of human PP1 *in vitro* (75). Further studies used a deletion mutagenesis approach to reveal that residues 286–296 of PP1γ were necessary and sufficient for PA-binding (76). Results from one study suggest that PP1 activity may play a role in suppressing T cell function in a rat model of alcohol intoxication and burn injury (77). While this suggests that PA-mediated inhibition of PP1 function may facilitate T cell activation, further experiments are required to better understand the role of PLD-derived and DGK-derived PA in suppressing PP1 activity in T cells.

Src homology region 2 domain-containing phosphatase 1 is a tyrosine phosphatase that plays a critical role in T cell function (78). “Moth-eaten” mice carry a spontaneous frame-shift mutation in the SHP1 gene and lack detectable SHP1 protein (79). Studies with these mice revealed a role for SHP1 in negatively regulating positive and negative thymocyte selection (80), while the use of conditional SHP1 knockout mice showed that SHP1 limits the number of short-lived effector CD8 cells produced in response to viral infection (81). Early studies showed that PA could increase the phosphatase activity of SHP1 toward the EGF receptor when the two proteins were transiently co-expressed in 293 cells (82). Subsequently, PA was shown to directly bind to recombinant SHP1, and two distinct PA-binding sites (a high affinity site on the C-terminal end and a low affinity site on the N-terminal end) were identified on SHP1 (83). Future studies are required to determine if PA modulates SHP1 activity in immune cells and if PA may serve as an effective therapeutic agent to modulate immune responses.

## Role of DGK Activity in Thymocyte Development

Bone marrow-derived early progenitor cells must go through an elaborate process of development in the thymus to become mature T cells (84, 85). Thymocytes at different stages of maturation are readily distinguished by a combination of CD4 and CD8 co-receptors expressed on their cell surface, proceeding from the earliest stage with neither CD4 nor CD8 (double negative/DN) through an intermediate stage expressing both CD4 and CD8 (double positive/DP) to a mature stage marked by the expression of either CD4 or CD8 (CD4 single positive/CD4SP or CD8 single positive/CD8SP) (86, 87). While progenitor cells enter the thymus at the cortico-medullary junction, a number of sequential chemokine/chemokine-receptor interactions help guide a developing thymocyte through the thymic cortex and medulla, facilitating its progressive relocation to appropriate micro-environments within the thymus (88, 89).

With the expression of RAG proteins, DN cells undergo VDJ recombination at the TCRβ locus, expressing a pre-TCR on the cell membrane. DN cells with a productive TCRβ rearrangement pass through the β-selection developmental checkpoint, undergoing multiple rounds of proliferation and upregulating expression of CD4 and CD8 to become DP cells. DP cells subsequently rearrange V and J genes at the TCRα locus, expressing a unique TCR on the cell surface. Subsequently, cells bearing TCRs that recognize self-peptide-MHC complexes on thymic epithelial cells receive survival signals during the so-called positive selection process, while others that fail to recognize these complexes die of “neglect” (90, 91). On the other hand, DP cells with TCRs that recognize self-peptide-MHC complexes with high affinity are eliminated by apoptosis-inducing signals during negative selection (92, 93). Together, positive and negative selection processes ensure the generation of a T cell repertoire that is both functional and self-tolerant (94, 95). DP cells that survive these selection processes mature into CD4SP and CD8SP cells that eventually migrate to secondary lymphoid organs as naïve CD4 and CD8 T cells (96, 97).

A plethora of studies have implicated DAG-dependent signaling pathways in β, positive and negative selection. For instance, early studies showed that signaling via the pre-TCR activates ERK1/2 (98), while more recent ones have demonstrated an essential role for RasGRP1 and ERK activation in efficient β-selection (27, 99). Mice with a T cell-specific deficiency of PLCγ1 show dramatically reduced numbers of mature CD4SP and CD8SP thymocytes, and defects in both positive and negative selection when crossed with HY TCR transgenic mice (100). Impairment of thymic selection in the absence of PLCγ1 suggests that its product DAG may play an important role in the process. Lending further credence to this notion, RasGRP1-deficient mice show impaired Ras-ERK signaling in thymocytes and defective thymic selection with a 70–90% reduction of mature SP cells (25, 26). Transgenic mice expressing a dominant negative form of Ras present with defects in positive selection, but not negative selection, when crossed with HY TCR transgenic mice (101). Similar observations were made with transgenic mice that expressed a catalytically inactive form of MEK1 (K97A) under the control of the thymocyte-specific Lck proximal promoter (102). ERK1 deficiency results in a severe developmental block at the DP stage (103). Conditional deletion of ERK2 using proximal Lck-Cre partially blocked DN3 to DN4 progression, while deletion with CD4-Cre led to defective positive selection. Mice with a combined deficiency of ERK1 and ERK2 showed that ERK activity is required for proliferation and differentiation associated with β-selection, and for positive selection (104). The MAP kinase-interacting serine/threonine kinases (Mnks) 1 and 2 lie downstream of ERK1/2 and p38 (105, 106). Recent studies have shown that TCR triggering can activate Mnk1/2 via the Ras-ERK pathway in a manner that is negatively regulated by DGK αζ activity (107). Although Mnk1/2 phosphorylate EIF4E, which is thought to promote translation initiation, combined deficiency of Mnk1/2 did not lead to obvious changes in thymocyte development. The mechanisms by which ERK1/2 regulate thymocyte selection remain to be clearly defined. Together, these studies suggest that the DAG-RasGRP1-Ras-ERK pathway plays a critical role in thymocyte development.

The role of the DAG-mediated PKCθ-IKK-NF-κB pathway in T cell development has also been studied extensively. While initial studies found no obvious developmental defects in PKCθ deficient thymocytes, more recent ones have suggested that PKCθ may be required for efficient positive selection (108, 109). T cell-specific deletion of IKKγ or replacement of IKKβ with a dominant kinase-dead form results in a reduction of mature CD8SP cells (110), while transgenic models that allow for activation or inhibition of NF-κB have revealed its role in the establishment of signaling thresholds for positive and negative selection (111).

The importance of these DAG-mediated pathways suggests that their tight regulation by DGK may be critical for normal thymocyte development. Studies with mice that lack both DGKα and DGKζ (DGKαζDKO) have confirmed this hypothesis (112). DGKαζDKO thymocytes experience excessive DAG accumulation and enhanced DAG-mediated signaling after TCR engagement. This is associated with a severe developmental block at the DP stage and a marked paucity of mature CD4SP and CD8SP cells. Defects in positive, but not negative, selection were revealed using a HY TCR transgenic system. Addition of exogenous PA to fetal thymic organ cultures increased the frequency of SP cells in DGKαζDKO thymi without obvious effects on control thymi, suggesting that DGKα and DGKζ play a synergistic role in T cell development not just by dampening DAG-mediated signals but also by promoting PA-mediated signals. This DGK-induced switch from DAG-driven to PA-driven signals may also play a critical role in preventing prolonged activation of the highly oncogenic Ras-ERK and NF-κB pathways in developing thymocytes. Indeed, HY TCR transgenic mice with decreased DGK activity showed significantly enhanced thymic lymphomagenesis, suggesting an important role for DGK activity in tumor suppression (112).

More recent work from our group has uncovered a novel role for DGKs as negative regulators of mTOR activity in thymocytes (113). Results from the study showed that low concentrations of phorbol 12-myristate 13-acetate (PMA), a functional analog of DAG, were able to induce phosphorylation of mTOR complex 1 substrates S6K1 and 4E-BP1 and mTOR complex 2 substrate Akt (S473), suggesting that DAG-mediated signaling is sufficient to induce activation of both mTOR complexes in thymocytes. DGKαζDKO thymocytes showed enhanced phosphorylation of S6K1, 4E-BP1, and Akt (S473) upon TCR engagement as compared to WT counterparts, suggesting that DGK activity inhibits TCR-induced activation of mTORc1 and mTORc2. Further studies are required to determine if dysregulated mTOR signaling might contribute to the defects in T cell development and function observed in DGKαζDKO mice.

Emerging evidence also suggests that tight regulation of DAG-mediated signaling by DGK activity may be critical for the development of *i*NKT cells. *i*NKT cells are a rare but distinct lineage of αβ T cells that express a highly restricted TCR repertoire and recognize glycolipids presented on CD1d. Sometimes called the “Swiss-Army knife” of the immune system, *i*NKT cells bridge innate and adaptive immunity by performing an array of functions that include killing of infected cells and secretion of cytokines and chemokines (114). Despite their relative rarity, an important role for *i*NKT cells has been demonstrated in immune responses to pathogens, allergens, self-antigens, and cancer.

Previous work has revealed a critical role for signaling via the PKCθ-IKK-NF-κB pathway in the ontogeny of *i*NKT cells (15, 115–117). More recent studies have identified that RasGRP1-Ras-ERK signaling may also be indispensable for *i*NKT development. In one study, the absence of RasGRP1 was associated with a severe reduction of *i*NKT cell numbers in the thymus, spleen, and liver (118). The generation of bone marrow chimeras showed that the *i*NKT cell developmental defects were cell-intrinsic, and the remaining RasGRP1-deficient iNKT cell population displayed both a selective absence of CD4^+^ cells and defects in TCR-induced proliferation. In another study, the expression of a dominant negative form of Ras dramatically hindered *i*NKT development (118, 119).

While lack of IKK-NF-κB and Ras-ERK signaling is detrimental to *i*NKT cell development, recent findings indicate that hyperactive signaling via these DAG-mediated pathways may also perturb *i*NKT development (120). In this study, T lineage specific expression of a constitutively active form of Ras resulted in a late stage block in *i*NKT cell maturation, and constitutively IKKβ activity was associated with increased cell death at multiple developmental stages. Since the maintenance of optimal levels of DAG-mediated signaling appears to be essential for normal *i*NKT development, we hypothesized that tight regulation of DAG-mediated signals by DGK activity might be essential for this process. Results from our studies showed that while *i*NKT cell numbers were unaltered in mice lacking either DGKα or DGKζ, they were dramatically diminished in DGKαζDKO counterparts, suggesting that these DGK isoforms may play a redundant role in regulating *i*NKT cell development (120). Defective DGKαζDKO *i*NKT development was associated with enhanced cell death and co-incident with enhanced activation of the Ras-ERK and NF-κB pathways. Taken together, these results suggest that DGKα and DGKζ work synergistically to maintain an optimal level of DAG-mediated signaling that is essential for normal *i*NKT development.

## Role of DGK Activity in T Cell Function

### DGKα and DGKζ in T cell activation

**Figure 2:**
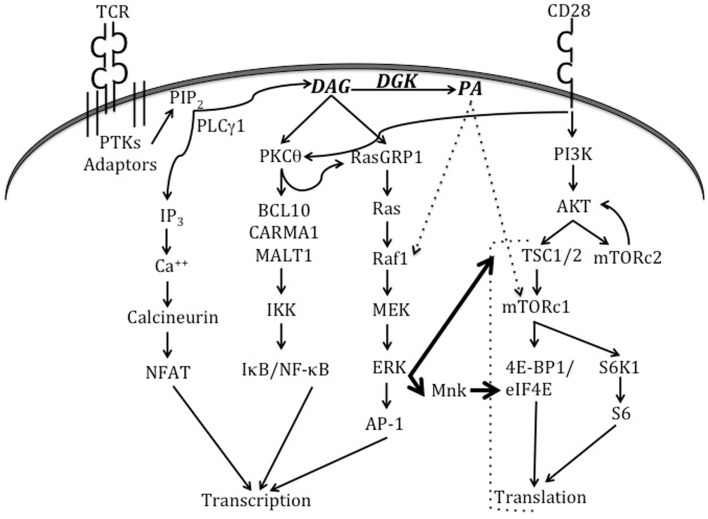
**Signaling pathways triggered by TCR and CD28 engagement**. When the TCR engages a cognate peptide-MHC complex in the presence of appropriate co-stimulatory signals, this activates TCR proximal tyrosine kinases (PTKs) and results in the recruitment of a number of adaptor molecules. Eventually, the activation of PLCγ1 enables it to hydrolyze membrane PIP_2_ to form second messengers IP_3_ and DAG. IP_3_ activates the calcineurin-NFAT pathway, while DAG activates the Ras-ERK-AP1 and NF-κB pathways. DGKs dampen DAG-mediated signals by converting DAG to PA. CD28 engagement plays an important role in the activation of PKCθ and the PI3K-Akt-mTOR axis. Recent work (indicated by thick arrows) has shown that TCR signaling can also directly activate mTOR complexes via the Ras-ERK pathway, and that such activation is negatively regulated by DGK activity. ERK can also activate Mnk1/2 kinases that phosphorylate eIF4E to promote translation. PA is produced in T cells by the action of both DGKs and PLDs (not shown in this figure). In other cell types, PA has been shown to activate Raf1 and mTORc1. Please refer to the text for more details about TCR-triggered signaling pathways and effector molecules that bind to DAG or PA.

T cell activation is a dynamic cellular process that involves the activation of multiple signaling cascades (Figure 2). The termination of such signaling, however, is important to prevent unrestrained immune responses and the development of autoimmunity. Research over the last decade has delineated a role for DGKα and DGKζ isoforms as molecular brakes that terminate DAG-mediated signals after TCR engagement. Early studies demonstrated that DGKζ is expressed in multiple lymphoid organs, with high levels in the T cell compartment (6). Overexpression of DGKζ in Jurkat T cells indicated that it substantially hindered TCR-induced Ras-ERK activation and upregulation of the activation marker CD69. DAG-binding and kinase domains, but not the ankyrin repeats, of DGKζ were found to be required for these inhibitory effects. Analysis of germline DGKζ knockout mice revealed no dramatic differences in T cell development or homeostasis (121). However, DGKζ-deficient T cells showed enhanced Ras-ERK activation and diminished PA production upon TCR engagement. Complementing observations from the Jurkat system, DGKζ-deficiency resulted in increased upregulation of CD69 and CD25 (markers of T cell activation) upon TCR engagement. Consistent with enhanced activation, DGKζKO T cells were hyper-proliferative in response to both antigenic stimulation and lymphopenia.

The role of DGKα in controlling T cell activation largely parallels that of its ζ counterpart. Overexpression of DGKα in Jurkat cells greatly impaired TCR-induced activation of a co-transfected AP1 driven luciferase reporter construct without obvious effects on calcium influx (122). While DGKαKO mice did not present with obvious changes in T cell development or homeostasis, enhanced Ras-ERK activation and hyper-proliferation were observed upon TCR engagement in DGKαKO T cells. Taken together, these results suggest that DGKα and DGKζ act in a non-redundant manner to restrain DAG-mediated signaling and prevent T cell hyper-activation upon TCR engagement. Further studies are required to better understand the unique mechanisms by which these isoforms act.

### DGKα and DGKζ in T cell anergy

Mechanisms of central and peripheral tolerance play a critical role in preventing the development of autoimmunity (123, 124). T cell anergy is a form of peripheral tolerance whereby T cells that recognize self-antigen in the absence of co-stimulatory signals are rendered functionally inactive (125–128).

While anergic T cells express elevated levels of DGKα as compared to naïve counterparts, levels of DGKζ increase or remain unchanged depending upon the experimental system (122, 129, 130). Transduction of resting Coxsackievirus and adenovirus receptor (CAR) transgenic T_H_1 cells with DGKα or DGKζ-containing adenoviral constructs revealed that overexpression of DGKα, but not DGKζ, was sufficient to cause an anergy-like state (130). T_H_1 cells transduced with the DGKα construct showed diminished ERK activation and IL-2 production in response to stimulation with anti-CD3 and anti-CD28. Experiments with RAG2-deficient 2C TCR transgenic cells also indicated that DGKα overexpression resulted in impaired recruitment of RasGRP1 to the plasma membrane. Brief treatment of anergic T_H_1 cells with a pharmacological inhibitor of DGK activity before overnight re-stimulation with anti-CD3 and anti-CD28 led to a dose-dependent increase in IL-2 production, suggesting a causal function for high DGK activity in T cell anergy. Similar results were obtained with RAG2-deficient 2C TCR transgenic T cells that had been rendered anergic *in vivo*.

Genetic evidence for the role of DGKα activity in T cell anergy comes from an *in vivo* anergy induction model in which mice were injected with the super-antigen staphylococcal enterotoxin B (SEB) that renders Vβ8^+^ T cells anergic (122). When re-stimulated with SEB *ex vivo*, in contrast to WT counterparts, Vβ8^+^ T cells from DGKαKO mice retained the ability to produce IL-2 and proliferate. These findings complement the data from the adenoviral-based over-expression studies, and confirm that DGKα is essential for anergy induction *in vivo*.

Further studies also indicate that DGKα and DGKζ may play synergistic roles in anergy induction (122). When splenocytes from WT, DGKαKO, and DGKζKO mice were depleted of CD8 cells and stimulated in the presence of anti-CD3 and CTLA4-Ig (to block co-stimulatory signals), DGKαKO, and DGKζKO T cells underwent 2–3 rounds of proliferation in contrast to WT counterparts that did not divide. Proliferation of DGKζKO T cells under similar culture conditions was highly enhanced by the addition of a DGKα inhibitor, and was comparable to that of WT cells stimulated with anti-CD3 and anti-CD28. These results support the notion that α and ζ DGK isoforms may act in a synergistic manner to induce T cell anergy.

### Role and regulation of DGKα and DGKζ at the plasma membrane

Early studies found that in T cell lines, DGKα translocated from the cytosol to the plasma membrane in response to stimulation via an ectopically expressed muscarinic type 1 receptor as well as via the TCR (131). Examination of the redistribution of GFP-tagged DGKα revealed rapid but transient translocation of cytosolic DGKα to the plasma membrane after anti-CD3 and anti-CD28 crosslinking. Tyrosine-kinase phosphorylation, along with increases in intracellular calcium levels, was found to be essential for receptor-induced membrane translocation of DGKα. Pre-treatment of cells with the type I DGK inhibitor R59949 enhanced DGKα translocation to the plasma membrane at 2 min but also prevented DGKα dissociation from the membrane even after 60 min, suggesting that removal of DAG to produce PA may play a critical role in enzyme release. Results from this study thus showed that plasma membrane localization of DGKα is controlled not just by receptor-derived signals, but also by its own enzymatic activity.

More recent work has suggested that direct tyrosine phosphorylation of DGKα by the Src family kinase Lck may promote its membrane association in T cells (132). Results from the study showed that Lck phosphorylates DGKα at the Y335 residue in the hinge region between its C1 domains and the kinase domain. TCR triggering was found to induce rapid and transient phosphorylation of DGKα at Y335 in both Jurkat cells and primary human T cells. Fractionation analysis of Jurkat cells revealed that Y335-phosphorylation was detected only in the membrane-associated (but not cytosolic) fraction. In addition, a Y335F mutant form of DGKα failed to show plasma membrane localization in response to anti-CD3/anti-CD28 stimulation, in contrast to its WT counterpart. Immuno-precipitation experiments showed that Lck and DGKα interacted with each other, but that the pool of DGKα pulled down with Lck was not phosphorylated at Y335. The authors hypothesize that Lck-mediated phosphorylation of DGKα may induce the latter’s dissociation from Lck but play a role in stabilizing DGKα at the membrane. Intriguingly, calcium flux induced by ionomycin was able to increase Y335-phosphorylation of DGKα, leading the authors to hypothesize that binding of Ca^++^ to DGKα’s EF hands might induce a conformation change that increases Lck-mediated phosphorylation in the basal state.

Another study has since shown that c-Abl, a tyrosine-kinase involved in regulating cell cycle and proliferation, directly phosphorylates DGKα at Y218 in NIH 3T3 cells (133). Phosphorylation of this residue is thought to play an important role in serum-induced export of DGKα from the nucleus to the cytosol, as a Y218F mutant form was not exported from the nucleus in response to serum addition.

A role for SAP (an adaptor molecule recruited by the SLAM family of co-receptors) in inhibiting DGKα activity following TCR/CD28 stimulation has also been identified recently (134). Results from the study showed that DGKα (but not DGKζ) activity was reduced in response to TCR/CD28 or TCR/SLAM stimulation in Jurkat cells and human peripheral blood lymphocytes. However, such inhibition was not observed in Jurkat cells upon shRNA-mediated knockdown of SAP. SAP knockdown was also found to impair the recruitment of DGKα to the plasma membrane selectively upon TCR/SLAM stimulation, but not TCR/CD28 stimulation, suggesting that the enzymatic activity and localization of DGKα may be regulated by distinct mechanisms. Overexpression of SAP was sufficient to reduce DGKα activity in Jurkat cells, providing further evidence of SAP’s role as a negative regulator. Such a role for SAP is also corroborated by findings from previous studies that SAP-deficient T cells showed reduced recruitment of PKCθ to the plasma membrane and diminished ERK1/2 activation upon TCR stimulation, leading to abnormal T cell differentiation and function (19). Pharmacological inhibition of DGKα activity by R59949 partially restored PKCθ membrane recruitment, ERK1/2 activation, and IL-2 production by SAP-deficient cells, suggesting that unrestrained DGKα activity might contribute to these signaling defects in the absence of SAP.

Like its α counterpart, the ζ isoform of DGK also shows dynamic changes in its subcellular localization in response to signals via the TCR. Early studies showed that GFP-tagged DGKζ rapidly translocated from the cytosol to the plasma membrane upon stimulation of an ectopically expressed muscarinic type 1 receptor, but not the TCR, in Jurkat T cells (135). Deletion of the C-terminal domain (containing the PDZ-binding domain and the ankyrin repeats), however, enabled DGKζ to translocate to the plasma membrane following TCR stimulation, suggesting that these domains may negatively regulate membrane translocation. The results also revealed that intact cysteine-rich C1 domains and PKCθ-mediated phosphorylation of the MARCKS domain are essential for DGKζ membrane translocation, while enzymatic activity is dispensable. Others have shown that DGKζ can translocate to the nucleus in COS-7 cells, and that this translocation is regulated by PKC-mediated phosphorylation of the MARCKS motif (136). Future studies should investigate a role for DGKζ in regulating nuclear DAG levels in immune cells.

When a T cell recognizes cognate peptide-MHC complexes and co-stimulatory molecules on an antigen-presenting cell (APC), this leads to the formation of a specialized junction at the T cell-APC interface. This so-called “immunological synapse” typically consists of a central cluster of T cell receptors surrounded by a ring of adhesion molecules, and synapse formation is thought to sustain robust signaling by facilitating the co-localization of kinases and adaptor proteins while excluding phosphatases (137, 138).

Previous studies have shown that DGKζ can physically associate with RasGRP1 in co-transfection experiments, but a similar function for DGKα was not tested (139). A recent study directly analyzed the recruitment of DGKα and DGKζ to the immunological synapse (140). Affinity purification of TCR complexes from Jurkat cells activated by anti-CD3 and anti-CD28 crosslinking suggested that both DGK isoforms were recruited rapidly to the TCR complex. However, video-microscopic experiments with GFP-tagged DGK proteins indicated that only DGKζ translocates rapidly to the plasma membrane at the early stages of synapse formation. These discrepant results need to be interpreted with caution, as fusion with GFP could potentially alter a protein’s structure and disrupt its normal localization pattern. However, RNA interference experiments from this study showed that PA production at the TCR complex was substantially reduced by knock down of DGKζ but not DGKα, strengthening the notion of functional differences between the isoforms. The addition of PMA was found to enhance DGKζ activity upon TCR stimulation, indicating that DAG itself may regulate DGKζ activity. The use of a fluorescently tagged DAG-sensor domain showed that both plasma membrane localization and kinase activity of DGKζ were critical for DAG consumption at the immune synapse. Together, these results indicate a specific function for the DGKζ isoform in regulating DAG levels at the immunological synapse. Further studies are required to fully characterize the TCR-induced translocation of DGKα and DGKζ in primary T cells.

### DAG and T cell secretion

The directed release of soluble factors is an important mechanism by which T cells kill target cells and communicate with other cell types. Early studies have shown that the microtubule-organizing center (MTOC) of a T cell reorients itself to a position just below the immunological synapse within minutes of TCR stimulation. Such polarization is though to aid in directional secretion by aligning the protein synthesis and secretion machinery of the T cell with the immune synapse (141, 142). Inhibiting MTOC translocation after TCR stimulation resulted in reduced phosphorylation of ZAP70 and LAT, disorganized immune synapse architecture and impaired IL-2 secretion, suggesting that MTOC translocation may also play a critical role in synapse formation and sustained TCR signaling (143).

More recent studies using a photoactivation system in which individual T helper cells can be activated by a pulse of ultraviolet light, have revealed a critical role for localized DAG production in MTOC polarization toward the synapse (144). MTOC polarization was abrogated in the presence of a PLCγ1 inhibitor, but unaffected in the presence of a Ca^2+^ chelator, suggesting that DAG may play a critical role in the process. Treatment with PMA, but not ionomycin, disrupted MTOC polarization providing further evidence that DAG, not Ca^2+^, links PLCγ1 activity to MTOC polarization. Data from imaging experiments showed robust accumulation of DAG-sensor proteins at the region of photoactivation, followed by reorientation of MTOC to this region with an average delay of 13 s between the two events. Treatment with a DGK inhibitor prevented sustained C1-GFP accumulation at the irradiated region and thereby impaired MTOC polarization. Experiments with a version of DAG that could be activated by ultraviolet light showed that a localized increase in DAG concentration was sufficient to drive transient polarization of the MTOC. Taken together, these experiments indicate an important role for DAG and DGKs in directional secretion. Treatment with PMA or a type II DGK inhibitor impaired T cell-mediated killing of target cells, without affecting degranulation. This suggests that localized DAG signaling plays a critical role in CTL killing not by blocking granule release but by directing the granules toward their appropriate target. Further studies using the photoactivation system have shown that DAG recruits three distinct PKC isoforms, ε, η, and θ, to the immune synapse to promote cytoskeletal polarization following TCR stimulation (145).

A role for DGKα has also been established in the secretion of lethal FasL-bearing exosomes during activation induced cell death (AICD) (146). In this study, pre-treatment with a type I DGKα inhibitor increased the secretion of FasL-bearing exosomes upon TCR stimulation, and enhanced FasL-dependent AICD in J-HM1-2.2 cell line, suggesting that DGK may act as a negative regulator of exosome secretion. Based on the co-localization of DGKα with the trans-Golgi network and its presence in secreted exosomes, the authors proposed a model by which DAG may recruit PKD1 to the trans-Golgi network to promote vesicle budding.

Building on these results, a more recent study has identified a role for DGKα in the polarization of multi-vesicular bodies (MVBs) involved in the secretion of FasL-bearing exosomes (147). MVBs are late endosomes containing multiple exosomes/vesicles within their lumen that are formed by inward budding of the limiting membrane. In this study, inhibition of DGKα activity with a type I inhibitor was found to increase the number of mature MVBs, while overexpression of DGKα inhibited their formation, indicating that DGKα may negatively regulate the formation of MVBs. However, siRNA-mediated inhibition of DGKα impaired the polarization of MVBs and subsequent exosome release, suggesting a positive role for DGKα in this process. Thus, DGKα plays a complex role in the secretion of FasL-bearing exosomes, impairing their formation but aiding their polarization toward the immune synapse.

### DGKα and DGKζ in CTL responses

CD8 responses or CTL responses are critical for host defense against intracellular pathogens and tumors. CTL responses typically consist of three distinct phases – an expansion phase during which antigen-specific CD8 cells proliferate rapidly and differentiate into effector cells that kill infected target cells, a contraction phase during which 90–95 percent of these effector CD8 cells undergo apoptosis in response to diminishing antigen levels, and a memory maintenance phase in which the remaining 5–10 percent of cells are retained as a small but stable pool of fast-responding memory cells (148–150). Much effort over the recent years has focused on how signaling mechanisms in CD8 cells can be manipulated to alter the amplitude and kinetics of the CTL response. Preliminary experiments with lymphocytic choriomeningitis virus (LCMV) infection showed that mice deficient in DGKζ mounted a more robust response to the pathogen than WT counterparts (121). DGKζ-deficient mice showed a greater increase of splenic CD8 cell numbers than WT mice at day 7, with a bigger portion of CD62L^lo^ CD44^hi^ effector-memory (T_EM_) cells and IFNγ-producing cells within the CD8 population. Viral titers were 50–70 percent lower in DGKζ-deficient mice than WT mice, arguing that DGKζ activity may negatively regulate CTL responses.

These results were confirmed and extended by a subsequent study, which showed that DGK activity differentially regulates primary and memory responses to LCMV (151). In this study, both DGKαKO and DGKζKO mice showed enhanced expansion and increased cytokine production upon LCMV infection, but contained fewer memory cells than WT counterparts after a 4-month period. When equal numbers of memory cells from these mice were transferred to new recipients and re-challenged with LCMV, DGK-deficient memory cells expanded significantly less than WT memory cells, indicating that DGK activity may somehow promote the expansion of memory cells. Other studies have revealed that the temporal kinetics of mTORc1 activity may play a critical role in effector versus memory differentiation of CD8 cells (152, 153). Results from these studies suggest that sustained mTORc1 activity may induce the expression of the T-box transcription factor T-bet that promotes effector differentiation. The identification of DGKs as negative regulators of mTOR activity (113) suggests the possibility that sustained mTORc1 activity in DGK-deficient CD8 cells might favor effector differentiation and mitigate memory formation. While mTORc1 activity was indeed found to be elevated in DGK-deficient CD8 cells (as measured by phosphorylation of the ribosomal protein S6) (151), further studies are required to dissect the contribution of enhanced mTORc1 activity to the dysregulation of CD8 responses seen in DGKαKO and DGKζKO mice.

DGKζ also acts as a negative regulator of anti-tumor CTL responses in an EL4-Ova lymphoma model (154). In this model, significantly smaller tumors were recovered from DGKζ-deficient mice as compared to WT mice, 3 weeks after implantation of tumor cells. Evaluation of CD8 splenocytes revealed a higher proportion of T_EM_ cells and a higher proportion of Ova-specific CD8 cells in DGKζ-deficient mice than in WT mice. An increased percentage of tumor-infiltrating CD8 cells was also found to be proliferating in DGKζ-deficient mice as compared to WT counterparts. Taken together, these results suggest that DGKζ activity may play a critical role in restraining anti-tumor responses, closely mirroring its functions during CTL responses to viral infection. When naïve WT-OT1 and DGKζKO-OT1 cells were adoptively transferred into congenically marked recipients that subsequently received EL4-Ova lymphoma cells, recipients with DGKζKO-OT1 cells developed smaller tumors. DGKζKO-OT1 cells also contained a bigger pool of CD44^hi^ cells and IL-2 producing cells. Collectively, these results argue for a CD8 cell-intrinsic role for DGKζ in curtailing anti-tumor responses.

Investigation of tumor-infiltrating CD8 cells in human renal cell carcinoma patients showed increased DGKα activity and diminished signaling via MAPK pathways, as compared to CD8 cells that were present in non-tumor areas of the kidney (155). Increased DGKα activity was associated with defects in granule exocytosis and lytic function of these CD8 cells, and treatment with a DGKα inhibitor was able to increase ERK phosphorylation. Culturing with low-dose IL-2 reduced DGKα expression and enhanced ERK activation and degranulation. IL-2 treatment also increased the frequency of tumor-infiltrating cells that produced perforin, granzyme B, or IFNγ. Taken together, these data indicate that increased DGK activity and DAG metabolism dampen the responsiveness of tumor-infiltrating CTLs in a reversible manner. What factors in the tumor microenvironment drive increased DGK activity in CD8 cells is an important question that remains to be addressed.

## Role of DGK Activity in Other Immune Cells

Apart from T cells, DAG and PA are critical signaling intermediates in several cell types including mast cells, dendritic cells (DCs), and macrophages. It is therefore not surprising that tight regulation of DAG and PA levels by DGK activity is essential for normal functioning of these cell types. Mast cells are abundant at the host’s interface with the environment, such as the skin and mucose (156). While best known for their role in the pathogenesis of asthma, allergy, and anaphylaxis, mast cells also play a critical role in pathogen surveillance and defense against parasites (157). In contrast to observations with T cells, mast cell function *in vivo* was diminished in the absence of the DGKζ, impairment of local anaphylactic responses (158). Bone marrow-derived mast cells that lacked DGKζ showed impaired degranulation but enhanced production of cytokines such as IL-6 when stimulated *ex vivo*. Other studies have shown that both the ζ1 and ζ2 isoforms of DGKζ expressed in bone marrow-derived macrophages and DCs, with ζ1 being predominant (159). Considered “professional phagocytes,” macrophages, and DCs express a diverse array of pattern recognition receptors (including toll-like receptors or TLRs) that enable them to detect the presence of pathogens and cell debris. Upon stimulation with *Toxoplasma gondii* – stable tachyzoite antigen (STAg, which activates multiple TLRs), DGKζ-deficient splenic DCs and bone marrow-derived macrophages (BMMϕ) produced less TNFα and IL-12 p40 than WT counterparts. Consistent with this impairment in cytokine production, both resistance to endotoxin shock and susceptibility to *T. gondii* infection were increased in DGKζ KO mice. While these findings provide tantalizing evidence of a role for DGKζ in regulating innate immune responses, further studies are required to gain a better understanding of the underlying molecular mechanisms. In addition, the involvement of other DGK isoforms in the regulation of innate immune responses remains to be investigated. The varied biological functions of DGK activity in T cells and other immune cells are summarized in Table 1.

**Table 1:** **Biological functions of DGKs in T cells and other immune cells**.

Functions regulated by DGK activity	Reference
DAG metabolism at the T cell-APC immunological synapse	Sanjuan et al. (131)
	Topham and Prescott (139)
	Santos et al. (135)
	Merino et al. (132)
	Baldanzi et al. (134)
	Gharbi et al. (140)
	Matsubara et al. (133)
Development of αβ T cells	Guo et al. (112)
	Gorentla et al. (113)
Development of *i*NKT cells	Shen et al. (120)
T cell activation and anergy	Zhong et al. (6)
	Zhong et al. (121)
	Olenchock et al. (158)
	Zha et al. (130)
CD8 T cell responses to pathogens and tumors	Zhong et al. (121)
	Riese et al. (154)
	Prinz et al. (155)
	Shin et al. (151)
MTOC polarization and directional secretion	Alonso et al. (146)
	Quann et al. (144)
	Alonso et al. (147)
Mast cell degranulation and cytokine production	Olenchock et al. (158)
Macrophage and DC cytokine production	Liu et al. (159)

## Summary

Over the past few years, a remarkable number of elegant studies that have furthered our understanding of the roles of DAG-mediated and PA-mediated signaling pathways, and their regulation by enzymes of the DGK family, in T cell development and function. A role for DGK activity has been identified in a variety of critical processes including conventional αβ T cell and *i*NKT cell development, T cell activation and anergy, directional secretion, and suppression of CD8 responses against viruses and tumors. While a multitude of interesting and fundamental questions in the field have been addressed by these recent studies, it is important to note that perhaps just as many others await answers. The roles of DGK-derived PA and PLD-derived PA in T cell development and function have proved challenging to dissect, as have differences between DGK isoforms in terms of substrate specificity and subcellular localization. Key elements such as transcription factors, microRNAs, and post-translational modifications that control the dynamic expression and function of DGKs during a T cell’s lifetime also remain relatively unexplored. Applying the scientific method to answer these intriguing questions is likely to yield a better understanding of how DAG and PA signals and DGK activity regulate immune responses, enhancing our ability to modulate such responses to quell self-reactivity or generate protective immunity.

## Conflict of Interest Statement

The authors declare that the research was conducted in the absence of any commercial or financial relationships that could be construed as a potential conflict of interest.
